# Detection of Respiratory Viruses in Deceased Persons, Spain, 2017

**DOI:** 10.3201/eid2407.180162

**Published:** 2018-07

**Authors:** Ana Navascués, Itziar Casado, Alejandra Pérez-García, Aitziber Aguinaga, Iván Martínez-Baz, Yugo Floristán, Carmen Ezpeleta, Jesús Castilla

**Affiliations:** Complejo Hospitalario de Navarra, IdiSNA, Pamplona, Spain (A. Navascués, A. Pérez-García, A. Aguinaga, C. Ezpeleta);; CIBER Epidemiología y Salud Pública, Madrid, Spain (I. Casado, A. Pérez-García, I. Martínez-Baz, Y. Floristán, J. Castilla);; Instituto de Salud Pública de Navarra, IdiSNA, Pamplona (I. Casado, I. Martínez-Baz, Y. Floristán, J. Castilla)

**Keywords:** Influenza, respiratory virus, respiratory syncytial virus, coronavirus, mortality, cause of death, viruses, Spain, respiratory infections

## Abstract

During the 2016–17 influenza season in Spain, we tested specimens from 57 elderly deceased persons for respiratory viruses. Influenza viruses were detected in 18% of the specimens and any respiratory virus in 47%. Only 7% of participants had received a diagnosis of infection with the detected virus before death.

Increases in all-cause deaths usually occur during annual influenza and respiratory syncytial virus (RSV) epidemics (*1**,**2*)*.* This excess is higher in seasons dominated by influenza virus A(H3N2) (*3**,**4*)*.* Ecologic design approaches have been used to estimate deaths caused by influenza and other respiratory viruses on the basis of weekly virus surveillance data (*1**–**5*). Because a small proportion of persons are tested for influenza virus before death (*6*)*,* the actual contribution of influenza to all-cause mortality is not well known. Other respiratory viruses are responsible for some influenza-like illnesses and related deaths (*7*) and have been related to deaths of unknown cause (*8*)*.* We conducted a pilot study to evaluate the feasibility of detecting influenza and other respiratory viruses in recently deceased persons and of estimating the prevalence of infections in persons who died within an influenza epidemic period.

## The Study

We performed this study in Navarre, Spain, during January 23–February 19, 2017, during the seasonal influenza epidemic (*9*)*.* Recruitment was conducted in 2 morgues by trained professionals. Persons >65 years of age who had died of natural causes regardless of the reported cause of death were included, after we obtained written informed consent from their closest relatives. We obtained nasopharyngeal swab specimens before the bodies were prepared for burial; we tested the swabs for influenza and RSV by reverse transcription PCR (RT-PCR). We tested negative samples for other respiratory viruses using multiple PCR (Allplex Respiratory Panel; Seegene, Seoul, South Korea).

We obtained demographic information and previous diagnoses from the epidemiologic surveillance system. We retrieved hospitalization and laboratory confirmation for respiratory viruses within the 30 days before the death from electronic healthcare databases. We obtained the underlying causes of death from the regional mortality register and grouped them into 5 categories according to the International Classification of Diseases, 10th Revision: neoplasms (codes C00–D49), nervous system diseases (codes G00–G99), circulatory system diseases (codes I00–I99), respiratory system diseases (codes J00–J99), and all other causes. We used the 2 tailed Fisher exact test to compare proportions.

The study period included the last 4 weeks of the 2016–17 influenza epidemic in Navarre, starting 2 weeks after the peak. This period was characterized by a high but descending number of hospitalizations of patients with laboratory-confirmed influenza and 27% excess in all-cause mortality (Technical Appendix Figure).

During the study period, 460 deceased persons >65 years of age were registered, 106 were attended in the participating morgues, and 57 (54%) were enrolled in the study. Nonparticipation resulted mainly from logistic problems and lack of signed consent.

Of the 57 participants in the study, 29 (51%) were women, 23 (40%) were <85 years of age, 50 (88%) had major chronic conditions, 5 (9%) had been resident in nursing homes, and only 12 (21%) had been hospitalized before death. Nonparticipants did not differ in these characteristics (Technical Appendix Table).

**Figure 1:**
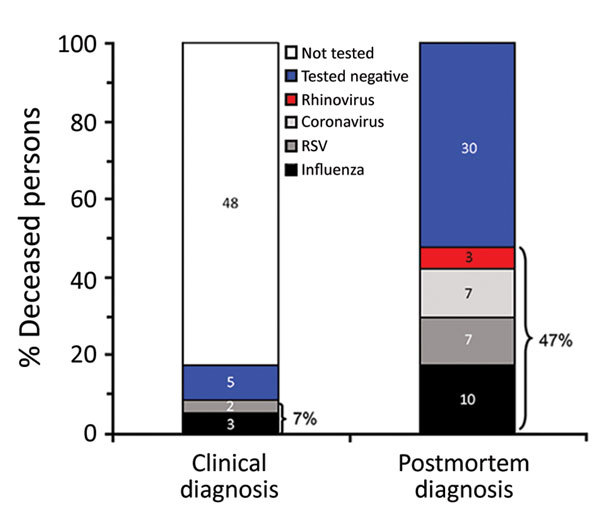
Clinical and postmortem detections of respiratory viruses among 57 deceased persons >65 years of age, Spain, 2017. As indicated, 47% of deceased patients tested positive for respiratory virus infection postmortem, but only 7% had received the same diagnosis before death. RSV, respiratory syncytial virus.

Respiratory viruses were detected in the postmortem study in 27 (47%) participants, but only 4 (7%) had received this diagnosis before death (Figure 1). Ten (18%) participants tested positive for influenza virus A(H3N2), 7 (12%) for RSV (4 subgroup A and 3 subgroup B), 7 (12%) for coronavirus (6 type 229E and 1 type OC43), and 4 (7%) for rhinovirus. Although postmortem detection of any respiratory virus was more likely among previously hospitalized persons, it was also frequent among those not hospitalized (75% vs. 40%; p = 0.050) (Table).

**Table:** Factors associated with postmortem detection of influenza and other respiratory viruses among deceased persons, Spain, 2017

Patient characteristics	Total no. patients	No. (%) patients	p value†

Influenza virus	Respiratory syncytial virus	Coronavirus	Rhinovirus	Any respiratory virus*
Total	57	10 (18)	7 (12)	7 (12)	4 (7)	27 (47)	
Sex							0.189
M	28	3 (11)	4 (14)	6 (21)	4 (14)	16 (57)	
F	29	7 (24)	3 (10)	1 (3)	0	11 (38)	
Age, y							0.889
65–74	7	2 (29)	0	0	1 (14)	3 (43)	
75–84	16	3 (19)	3 (19)	1 (6)	0	7 (44)	
>85	34	5 (15)	4 (12)	6 (18)	3 (9)	17 (50)	
Major chronic conditions‡	50	8 (16)	7 (14)	6 (12)	4 (8)	24 (48)	1.000
Nursing home residence	5	1 (20)	0	1 (20)	1 (20)	2 (40)	1.000
Hospitalization before death	12	3 (25)	2 (17)	2 (17)	3 (25)	9 (75)	0.050
Premortem diagnosis	4	3 (75)	1 (25)	0	0	4 (100)	0.044
Cause of death							
Neoplasms	16	1 (6)	1 (6)	4 (25)	0	6 (38)	0.391
Nervous system condition	8	2 (25)	0	1 (13)	0	3 (38)	0.709
Circulatory system condition	14	3 (21)	2 (14)	0	1 (7)	6 (43)	0.765
Respiratory system condition	7	2 (29)	3 (43)	0	1 (14)	6 (86)	0.045
Other causes	12	2 (17)	1 (8)	2 (17)	2 (7)	6 (50)	1.000

*One person’s specimen tested positive for both coronavirus and rhinovirus. †The 2-tailed Fisher exact test was used to compare the proportions of patients with any respiratory virus infection for the listed variables. ‡Major chronic conditions included heart disease, respiratory disease, renal disease, cancer, diabetes mellitus, cirrhosis, dementia, stroke, immunodeficiency, rheumatic disease, and morbid obesity.

**Figure 2:**
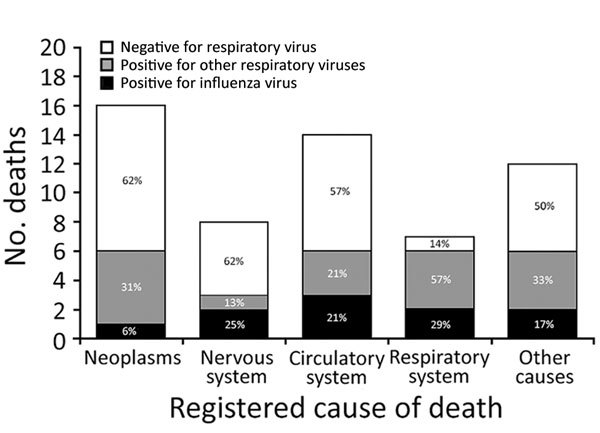
Postmortem detection of influenza and other respiratory virus infection by underlying cause of death among 57 deceased persons >65 years of age, Spain, 2017.

The postmortem detection of influenza or other respiratory viruses was not statistically associated with the analyzed covariates, with 2 exceptions: respiratory viruses other than influenza were detected more frequently in deceased men (46%; 13/28) than in women (14%; 4/29; p = 0.010), and respiratory viruses were more frequently detected among deceased persons who were reported with respiratory system diseases as the underlying cause of death than in those reported with other causes (86% vs. 42%; p = 0.045) (Table). Nevertheless, the percentage of deceased persons whose specimens tested positive for any respiratory virus was notable in all groups of nonrespiratory causes of death (range 38%–50%) (Table; Figure 2). Only 1 person (10%) whose specimen was detected as having influenza virus in the postmortem test had influenza registered as the cause of death; 5 (50%) were registered as having a cardiorespiratory cause of death.

## Conclusions

This study demonstrates the feasibility of the detection of respiratory viruses in samples from deceased persons. Respiratory viruses were found in nearly half of the persons who died of natural causes in an influenza epidemic period, and 18% were confirmed for influenza virus A(H3N2), which was the same influenza virus subtype that dominated in patients during the 2016–17 season (*10**,**11*)*.* The 2016–17 influenza season was characterized in Europe by an increase in deaths (*2*)*.* Other respiratory viruses were detected during the influenza circulation period and may have contributed substantially to hospitalizations and deaths (*1**,**5**,**12*)*.* RT-PCR seems to have high sensitivity for the detection of respiratory viruses in deceased persons, as previously shown in studies based on coronial autopsies (*8**,**13*).

Respiratory virus infections are characterized by sudden onset; death may occur suddenly, even before the symptoms are evident. Respiratory viruses can trigger secondary bacterial infections or exacerbate existing chronic conditions, and these concurrent conditions usually prevail as the underlying cause of death. Half of the deaths with influenza virus detection in the postmortem test were registered as having a noncardiorespiratory cause of death, which is consistent with a previous hospital study (*6*). This finding demonstrates the difficulty in estimating the deaths related to respiratory viruses by using the mortality registers.

These results raise relevant implications. Only a small proportion of deceased persons whose respiratory virus was detected in the postmortem test had been hospitalized and received this diagnosis before dying; therefore, the contribution of viral infections to death may be underestimated. Deaths related to respiratory viruses could be distributed among all causes of death. Although the burden of death has been estimated by indirect approaches (*1**–**5*), this study offers a complementary novel approach to assess the impact in terms of the proportion of all-cause deaths with respiratory virus detection (*14*).

The surveillance of influenza based on laboratory-confirmed cases is implemented in primary healthcare and in hospitalized patients (*9**,**11*). Our results open the possibility and show the potential interest of adding a sentinel virological surveillance based on persons who die during the influenza season.

Caution should be paid in the interpretation of these results, however. Virus detection does not necessarily imply a causal relationship between virus infection and death because respiratory viral shedding has been described in asymptomatic persons (*15*). Our study included 12% of deaths in the region during the last 4 weeks of the influenza epidemic, but the peak was not included; therefore, the representativeness is limited. Similar characteristics of participants and nonparticipants rule out selection bias. We cannot rule out false-negative results, however, because samples were obtained postmortem and the time from symptom onset to swabbing was unknown. Only negative samples for influenza and RSV were tested for other respiratory viruses, which might underestimate the frequency of the other respiratory codetections.

In summary, we demonstrate the feasibility of detecting respiratory viruses in recently deceased persons. We frequently detected respiratory viruses postmortem in winter deaths, although most of these infections were not clinically diagnosed. Respiratory virus surveillance systems could be complemented by testing persons who die during the influenza circulation period for respiratory virus infections.

Technical Appendix Additional information about the participants in the study of postmortem detection of respiratory viruses, Spain, 2017.
