# Supplementary material for: Detection of Respiratory Viruses in Deceased Persons, Spain, 2017
Source: Emerg Infect Dis. 2018 Jul;24(7):1331–4. doi: 10.3201/eid2407.180162 (PMC6038767; doi:10.3201/eid2407.180162)
Supplement: Technical Appendix — Additional information about the participants in the study of postmortem detection of respiratory viruses, Spain, 2017. [file 18-0162-Techapp-s1.pdf]

Article DOI: https://doi.org/10.3201/eid2407.180162

Detection of Respiratory Viruses in Deceased Persons, Spain, 2017

Technical Appendix

Viral Culture of the Influenza-Positive Samples

Samples that tested positive for influenza were tested in shell vial MDCK cell culture. Cell culture for influenza virus was successful in 2 of the 10 RT-PCR positive samples. The detection of influenza virus was poor in the cell culture, which may be attributable to the time since the infection or to the decline in viral viability postmortem.

Technical Appendix Table 1. Characteristics of the participants and nonparticipants in the study of postmortem diagnosis of respiratory virus, Spain, 2017.

Characteristic	Participants N = 57	Nonparticipants* N = 49	p value†
Date of death, 2017			0.789
Week 4	14	16	
Week 5	15	10	
Week 6	14	12	
Week 7	14	11	
Women	29 (51%)	23 (47%)	0.702
Nursing home residence	5 (9%)	3 (6%)	0.723
Age $\geq$85 y	34 (60%)	26 (53%)	0.558
Previous hospitalization	12 (21%)	12 (24%)	0.817

*Nonparticipants were persons who were not enrolled because of diverse circumstances: more than 24 h since the death, legal issues, logistic problems, a trained professional not available, close relative not available before preparing the dead body, and lack of signed consent.
†Two-tailed Fisher exact test was used to compare proportions.

Technical Appendix Table 2. Threshold cycle values and cell culture in samples with positive result for influenza virus by reverse transcription PCR, Spain, 2017

Participant no.	Influenza type	Subtype	Threshold cycle (Ct) value	Cell culture*	Premortem test result for influenza
1	A	AH3	30	Negative	Not available
2	A	AH3	31	Negative	Positive
3	A	AH3	30.65	Negative	Positive
4	A	AH3	32	Negative	Not available
5	A	AH3	33	Negative	Not available
6	A	AH3	25	Negative	Not available
7	A	AH3	19.96	Positive	Not available
8	A	AH3	29	Positive	Not available
9	A	AH3	23.87	Negative	Not available
10	A	AH3	35	Negative	Not available

*Evaluated by indirect immunofluorescence.

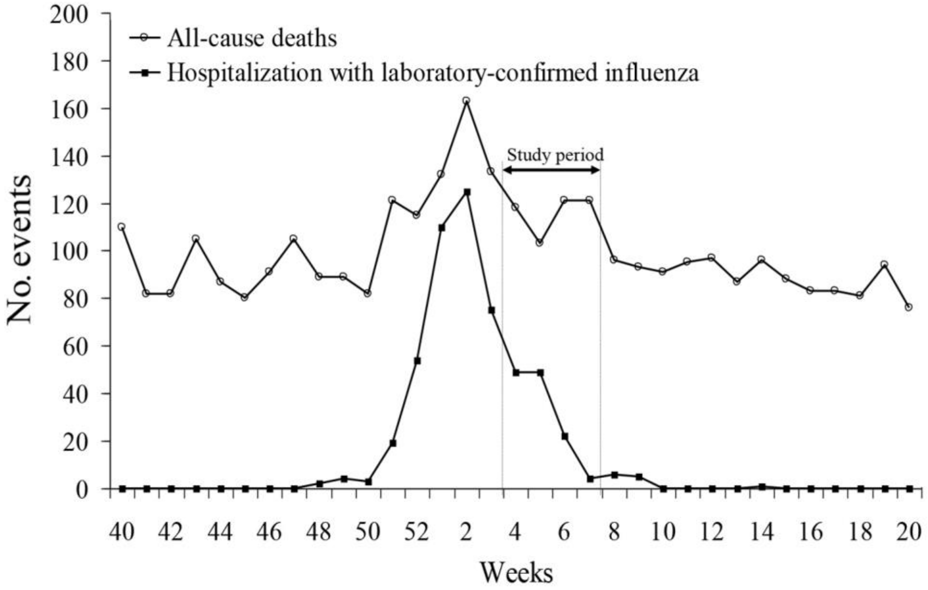

Technical Appendix Figure. Weekly incidence of hospitalized cases with laboratory confirmed influenza and all-cause deaths in persons ≥65 years of age in Navarre, Spain, during the 2016–17 influenza season. Study period was during weeks 4–7, 2017.
